# Quantitative Systems Pharmacology can reduce attrition and improve productivity in pharmaceutical research and development

**DOI:** 10.3389/fphar.2014.00247

**Published:** 2014-11-10

**Authors:** Tarek A. Leil, Richard Bertz

**Affiliations:** Clinical Pharmacology and Pharmacometrics, Exploratory Clinical and Translational Research, Bristol-Myers Squibb, Princeton, NJ, USA

**Keywords:** Quantitative Systems Pharmacology, pharmaceutical R&D, modeling and simulation, Systems Biology, pharmacometrics, drug discovery and development

## Abstract

The empirical hypothesis generation and testing approach to pharmaceutical research and development (R&D), and biomedical research has proven very effective over the last half-century; resulting in tremendous increases productivity and the rates of approval for new drug applications at the Food and Drug Administration (FDA). However, as discovery of new therapeutic approaches for diseases with unmet medical need becomes more challenging, the productivity and efficiency of the traditional approach to drug discovery and development is diminishing. Innovative approaches are needed, such as those offered by Quantitative Systems Pharmacology (QSP) modeling and simulation. This “systems” approach to modeling and simulation can be used to guide the hypothesis generation and testing process in pharmaceutical R&D, in a manner similar to its adoption in other industries in the past. Embedding QSP into the existing processes within pharmaceutical discovery and development will be required in order to realize the full beneficial impact of this innovative approach.

## INTRODUCTION

**FIGURE 1:**
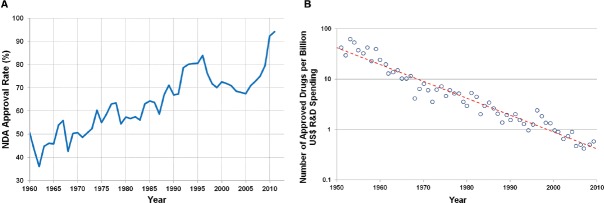
**(A)** Rate of approval for NDAs since 1960 (U.S. Food and Drug Administration, 2013b). **(B)** Number of approved drugs for every billion US dollars spent on R&D (adjusted for inflation; Scannell et al., 2012).

One of the oldest documents recording the process of drug discovery is the Ancient Egyptian “materia medica” dating to the sixteenth century B.C. This approach to drug discovery, based on empirical evidence from the natural world, is known as Pharmacognosy (de Pasquale, 1984), and was the primary means of drug discovery until the middle of the twentieth century. Advances in biochemistry, molecular and cellular biology, and medicinal chemistry during middle of the twentieth century resulted in a shift to a more hypothesis driven, mechanism-based approach to drug discovery (Drews, 2000). This approach includes mining of data from human epidemiology studies, combined with non-clinical *in vitro* and *in vivo* experiments to demonstrate the validity of a therapeutic target. The addition of high throughput chemical synthesis and screening permits identification of target selective, high affinity compounds. This hypothesis driven approach to pharmaceutical research and development (R&D) has been a tremendous advance relative to the ancient methods of Pharmacognosy, and resulted in a dramatic increase in the percentage of new drug applications (NDAs) approved by the Food and Drug Administration (FDA) since the early 1960s (Figure 1A; U.S. Food and Drug Administration, 2013b). However, there appears to be a decrease in the productivity (Figure 1B) of this approach that appears to be due to at least two major factors. One being the increasing difficulty in finding novel therapeutic targets, either for diseases with well established standards of care or those with unmet medical need (Pammolli et al., 2011; Scannell et al., 2012), and the other being the increasing cost associated with discovery and drug development of new drugs. The current average cost to bring a drug to market is $1.5 billion, over 10 times higher than the cost in the 1970s (DiMasi and Grabowski, 2007; Scannell et al., 2012). The need for improved productivity in the pharmaceutical industry has been recognized by the FDA, with the establishment of its “Critical Path Initiative” in 2004. This initiative was intended to improve the drug and medical device development processes, the quality of evidence generated during development, as well as the outcomes of clinical use of these products (Woodcock and Woosley, 2008).

## COMPUTER MODELING AND SIMULATION AS TOOLS TO IMPROVE PRODUCTIVITY

A decline in productivity is to be expected for any industry as it matures, and the competition from established products increases. This decline in productivity is typically associated with increasing development costs, partly due to the difficulty in differentiating one product from another in the marketplace. Industries must find innovative ways to increase the probability of commercial success while at the same time decreasing development costs. Most industries eventually realize the value of computer aided modeling and simulation as one of the means for achieving both of these objectives. Computer aided modeling and simulation allows testing of numerous potential scenarios “*in silico*” to eliminate those associated with a low probability of success, avoiding the tremendous costs of evaluating all of those failed scenarios in the real world. Today, aerospace, automotive, electronics, and other industries routinely incorporate modeling and simulation into their R&D processes (Woltosz, 2012). The pharmaceutical industry has been slow to integrate computer aided modeling and simulation for many reasons: including the perception that biology and pharmacology are “too complex” to be modeled with mathematical equations; a lack of adequate graduate training programs for pharmaceutical modeling and simulation scientists; and the lack of support from government funding agencies for academic research in computer aided modeling and simulation approaches for biomedical research. However, in the last decade, both the FDA and National Institutes of Health (NIH) have recognized the value of modeling and simulation in increasing productivity in biomedical research and pharmaceutical R&D. The FDA established its Pharmacometrics Division in 2009 to promote and evaluate the use of modeling and simulation approaches in regulatory submissions to the agency (U.S. Food and Drug Administration, 2013a). In 2011, the NIH published a white paper describing the emerging discipline of Quantitative Systems Pharmacology (QSP) modeling, and recommended the establishment of NIH-supported interdisciplinary research and training programs for QSP (Sorger et al., 2011). QSP modeling and simulation is a new term to describe the integration of two disciplines that have been increasingly useful in biomedical research and pharmaceutical R&D; “Systems Biology” and “Quantitative Pharmacology.” Systems Biology is the field of biomedical research that seeks to characterize biological networks of interactions, including those between genes and biologically active molecules to develop models of these systems that are usually qualitative in nature. Quantitative Pharmacology (a.k.a. Pharmacometrics) is the field of biomedical research that seeks to use computer aided modeling and simulation to increase our understanding of the pharmacokinetics (PK) and pharmacodynamics (PD) of drugs, and to aid in the design of pre-clinical and clinical experiments. The purpose of QSP modeling is to develop quantitative computer models of biological systems and disease processes; as well as the effects of drug PK and PD on those systems.

## PHARMACEUTICAL R&D AND QUANTITATIVE SYSTEMS PHARMACOLOGY

**FIGURE 2:**
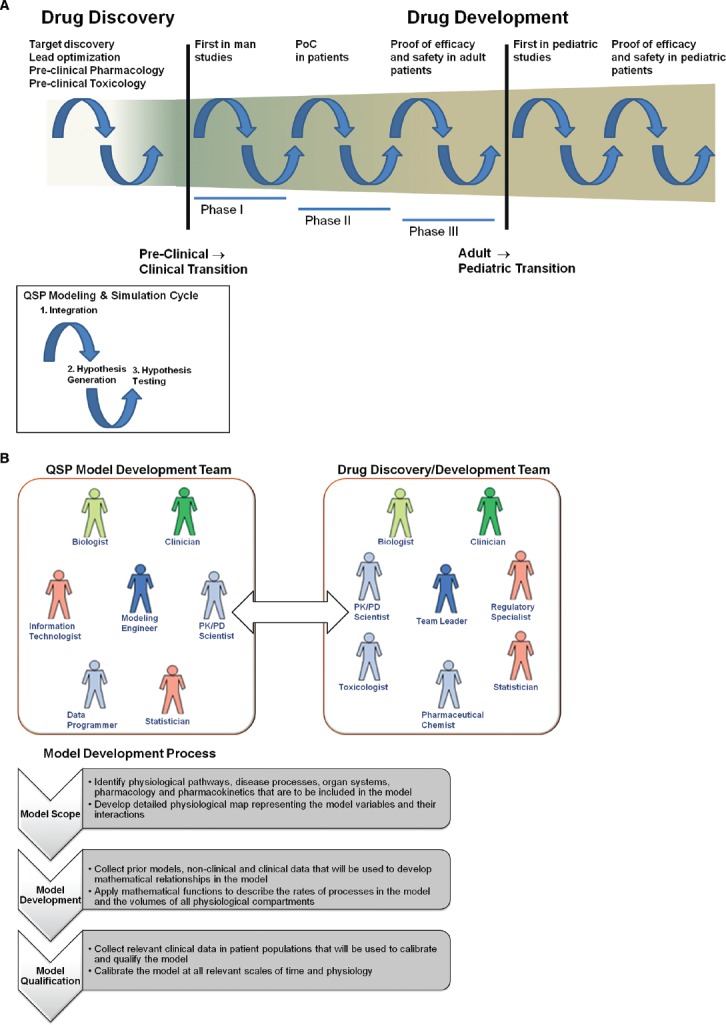
**(A)** Incorporation of Quantitative Systems Pharmacology modeling and simulation in pharmaceutical R&D. Drug discovery and development is a long and complex process with numerous transition periods where effective translation from one experimental model to the next is a challenge. Major transitions occur when moving to first in man studies and first in pediatric studies. The cycles of application of QSP modeling and simulation are defined by (1) integration of experimental data and biological knowledge to develop QSP models; (2) generation of hypotheses for potential outcomes in future experiments; (3) testing of those hypotheses with experiments that have been designed via simulation from QSP models. **(B)** Integration of QSP models in pharmaceutical R&D process. The model development team should be a sub-team of existing drug discovery and development teams. The goals of the model development team are to develop QSP models for their particular disease area and/or to apply existing QSP models to facilitate key milestones in discovery and development. The model development process should be rigorous and stepwise, so that models that are developed can used broadly in the disease area and can be used to communicate with regulatory agencies.

Pharmaceutical R&D is a stepwise process where investment in further characterizing the pharmacology of a candidate molecule is incrementally increased as confidence in the molecule’s probability of regulatory and commercial success increases. Investment initially starts with *in vitro* biochemical and pharmacology studies; then moves to animal pharmacology and toxicology studies, then to human healthy volunteer pharmacology and toxicology studies; and finally to large and expensive patient efficacy and safety studies. QSP models are based on the fundamental understanding of biological pathways, disease processes, and drug mechanisms of action. Therefore, they are very effective tools for integration of prior collected biological/pharmacological knowledge, formulation of pharmacological hypotheses, and for efficient translation between the various experimental models within pharmaceutical R&D. Key milestones in R&D where QSP models will be critical to increasing the probability of success will be in the target identification stage, the transition from pre-clinical to first in man studies, the transition from healthy volunteer to patient studies, and the transition from adult to pediatric (Figure 2A). These milestones represent the point where knowledge from one set of experimental models (e.g., animal) must be effectively translated to another set (e.g., human). QSP can facilitate this translation by formal integration of the knowledge from the original experimental model and generation of hypotheses for potential outcomes in the next experimental model. Computer aided simulations to guide the design of experiments intended to test those hypotheses.

In addition to their utility in translation between experimental models, QSP allows prediction of the effects of multiple therapeutic interventions in combination. As it becomes clear in many therapeutic areas that modulation of single drug targets is less effective (e.g., oncology, virology), the cost of testing all of the potential combinations in the clinic is prohibitive. QSP can provide a framework in which to evaluate these potential combinations prior to testing in the clinic, by providing a fundamental systems and quantitative understanding of how these different mechanisms will interact.

## PHARMACEUTICAL R&D AND QUANTITATIVE SYSTEMS PHARMACOLOGY

Published examples describing the use of QSP modeling and simulation to facilitate biomedical research and pharmaceutical R&D have been increasing in recent years. Most of these publications have been focused on PK, since the processes that govern drug absorption, distribution, metabolism, and excretion are better established compared to those that govern disease biology and PD (Edginton et al., 2008). Strougo et al. (2012) demonstrated the use of these physiologically based PK (PBPK) models for prediction of PK in children prior to the conduct of the first pediatric clinical studies. There are software packages that can be licensed with PBPK models incorporated that allow prediction of *in vivo* drug PK based on the *in vitro* properties of the molecule (Kuentz et al., 2006; Jamei et al., 2009). QSP models that predict both PK and PD are much more complex, and tend to be disease area specific. Vega-Villa et al. (2013) published a QSP model of the nitric oxide metabolic pathways and demonstrated the models ability to predict toxic methemoglobin levels in humans treated with nitric oxide. Geerts et al. (2013a) published a QSP model of cognitive deficit in schizophrenia and were able to simulate the enhancement of cognition with clozapine and risperidone, as well as the worsening of cognition with γ-aminobutyric acid (GABA) modulators lorazepam and flumazenil. There are software packages that can be licensed that allow prediction of both PK and PD for a variety of drugs and mechanisms of action, but at much greater expense compared with those used for PBPK alone (Shoda et al., 2010; Eissing et al., 2011). Agoram and Demin (2011) reported on the development and application of a QSP model of the PD of the 5-lipoxygenase (5-LO) pathway. This QSP model has been used to explain the complex PK–PD relationship of zileuton, a marketed 5-LO inhibitor (Karelina et al., 2010). The model was able to demonstrate the mechanism behind the longer duration, but similar magnitude, of action with the 600 vs. 400 mg dose of zileuton. More important for its utility in drug discovery, this QSP model could be used to predict the PK and PD of a new molecule or combination of molecules intended to modulate another component of the 5-LO pathway based solely on the biophysical properties of the molecule and its potency at the target. One could then design a series of *in vivo* experiments to validate the hypotheses generated from these predictions.

Quantitative Systems Pharmacology holds great promise in being able to uncover innovative therapeutic paradigms for complex multi-factorial diseases such as Alzheimer’s and diabetes. Because these diseases involve multiple physiological processes and can affect multiple organs, QSP can provide an integrated understanding of the pathology as well as the possible complex counter-intuitive results of therapeutic intervention.

## INTEGRATION OF QUANTITATIVE SYSTEMS PHARMACOLOGY INTO PHARMACEUTICAL R&D

In order to leverage QSP to accomplish this, it must be properly integrated into the decision making process in pharmaceutical R&D. As mentioned above, it is possible to license PBPK and QSP models to facilitate decision making in pharmaceutical discovery and development. However, since licensing is often limited to a few specialized functions, this approach decreases the flexibility and utility of QSP across the different functions within R&D. QSP models should be integrated into the processes of discovery and development within pharmaceutical companies in order to maximize their potential benefit on R&D efficiency and productivity. To better integrate these models into the existing processes, they can be developed internally within the drug discovery and development teams. In addition, the teams must be organized and educated to support this integration. Figure 2B shows the integration of the QSP model development team with the drug discovery and development team. Development of a QSP model for prediction of clinical PK and PD should be a rigorous and stepwise process, with three main steps:

(1) **Model Scope:** Development of the scope of the model, with delivery of a physiological pathway map representing all of the biological/pharmacological processes that will be incorporated in the model;
(2) **Model Development:** Prior models, relevant non-clinical and clinical data are collected to inform the incorporation of the mathematical equations that describe the processes and compartment volumes in the model;
(3) **Model Qualification:** The model is calibrated to relevant data from the target patient populations.

In addition to the modeling engineer, who will incorporate the equations into the model based on the agreed upon scope, critical individuals on the model development team include the following:

• **Biologist and Clinician:** Guide the scope of the model and inform the integration of relevant biology/pharmacology into the model, as well as identifying non-clinical and clinical data to be used in model development and qualification;
• **Data Programmer:** Curates and maintains the clinical/non-clinical databases that serve as inputs to the model;
• **Statistician:** Guide analysis of the simulation outputs and how they are used to inform the design of future clinical trials; the modeling engineer will incorporate the mathematical equations in the model and calibrate the model to relevant clinical data;
• **Information Technologist:** Maintain the software interface to the model and develop software for simulation;
• **PK/PD Scientist:** Provide broad expertise on the PK and PD aspects of the model and will serve as the primary mediator between the model development team and drug discovery and development team.

Once a QSP model is developed, it should be regularly updated with relevant internal and external research. In addition, it should be made available to all scientists in R&D that work in that particular disease area. The QSP model can be made flexible such that it can be readily adapted to other species that may be of interest in drug discovery (e.g., rat, monkey, rabbit, etc.). This would facilitate translation of experiments between these species and human.

## OUTLOOK FOR QUANTITATIVE SYSTEMS PHARMACOLOGY IN PHARMACEUTICAL R&D

Quantitative Systems Pharmacology holds great promise in being able to uncover innovative therapeutic paradigms for complex multi-factorial diseases such as Alzheimer’s and multiple sclerosis. Because these diseases involve multiple physiological processes and can affect multiple organs, QSP can provide an integrated understanding of the pathology as well as the possible complex results of therapeutic intervention. QSP thus offers pharmaceutical R&D an innovative way to conduct at drug discovery and development, particularly in diseases that are poorly translated from animal disease models. Geerts et al. (2013b) recently published an article on how QSP, when combined with phenotypic screening and preclinical animal models, could be used to address the bottleneck in both cognitive and neuropsychiatric drug discovery and development for Alzheimer’s disease. For such complex diseases that are poorly translated from animal disease models, target-focused drug discovery holds little promise for finding successful therapies. However, pharmaceutical companies are large and bureaucratic, and dramatic changes to the direction in which R&D is conducted may be adopted rather slowly.

There are smaller pharmaceutical and biotech companies that are fully integrating QSP into their biomedical R&D processes. One example is Merrimack Pharmaceuticals in Cambridge, MA, USA; founded by MIT professor of Biology and Biological Engineering Michael Yaffe. Merrimack states on their website, “We are a Systems Biology company. We believe that improving cancer care requires a systems-based understanding of the dynamic interactions within a cancer cell and its environment” (Merrimack Pharmaceuticals Inc., 2014). The scientific and financial communities will be watching these small companies, and if their model for a systems-based approach to R&D is successful, the pressure will be increased for “large pharma” to more fully adopt such innovative approaches into their discovery and development processes.

### Conflict of Interest Statement

The authors are employees of Bristol-Myers Squibb.
